# The New and the Old

**Published:** 1892-03-26

**Authors:** 


### The Hospital

AN INSTITUTIONAL JOURNAL OF

Science, Medicine, Nursing, and Philanthropy.

*For Hospitals, Asylums, Infirmaries, Dispensaries, Medical Practitioners, Students, Nurses, and the Charitable Public.*

VOL. XI.—No. 287.] [MARCH 26, 1892.

### THE NEW AND THE OLD.

It is a circumstance which presses itself upon general attention that whilst many of the finer spirits of our time stand by the old in politics and religion, in medicine all classes of minds have a decided preference for the new. There are some who go so far as to believe that all the physicians and surgeons who practised earlier than the second half of the nineteenth century were, to a large extent, ignorant of science, and mere imitators of confident predecessors, more ignorant even than themselves. That, of course, is an entirely absurd view of the facts, and can only be held by very young or very foolish persons. Nobody, however, can fail to be struck by the assumption of infallibility which is characteristic of senior medical students and newly-fledged practitioners, and of the extreme readiness of the less-instructed members of the general population to take those medical fledglings at their own valuation.

There is no doubt a reason for this somewhat unusual attitude of mind which is so universally taken towards the medical art. Such a mental state seems to contradict the natural conservatism of ancient races. But the contradiction is only apparent, and not by any means real. What men usually want to conserve is not institutions in the abstract, but themselves, and institutions so far as they appear to conserve the interests of those who strive to maintain them. On this hypothesis it is easy to understand why men elect to put their trust in the new medicine rather than in the old. They do not look upon the medical art with eyes of sentiment; they regard it from the purely utilitarian point of view. They pin their faith to those methods which they believe to be most conservative of life. Herein, though they choose the new rather than the old, and because they choose the new, they are acting in strict accordance with the instincts of a rational and worthy conservatism.

But the public are not quite the most competent of judges on this question of the new and the old in medicine; neither, indeed, are all the members of the medical profession. The public follow the fashions and the most imperious voices of the times in medicine as in other things; and it cannot be doubted that the ordinary medical practitioner follows the lead of a dozen or a score of the master spirits of his class with a docility almost amounting to slavishness. This somewhat excessive loyalty has its commendable as well as its unfortunate aspects. It is certain that the recognised leaders of medicine, perhaps especially of British medicine, at the present time, are broadly intelligent, unprejudiced, and exceedingly capable men. They have everything to gain and nothing to lose by making the best possible use of the science of their time, and of the innumerable practical methods which have had their origin in a competent empiricism. So thinking, and so acting, they are worthy both of confidence and of imitation. But nothing can compensate a man for the loss of his own proper originality of judgment, and just self-reliance. Merely to imitate any person, however distinguished, is to ensure for the imitator a habit of mental feebleness, which sooner or later, like a sword with a flaw in it, will fail him at what may be a life and death crisis. The least known and least self-reliant of practitioners should preserve his independence of mind at any cost.

No really thoughtful man can for a moment believe that all the science and all the competent practice have had their origins in the present generation. Comparing medicine with other sciences and arts we are bound to conclude either that physicians and surgeons have for five thousand years been the least intellectual, and the least worthy of all human creatures, or that, as in politics, religion, and law, there have been great leaders in former generations; leaders whom even the men of the present may fittingly reverence; and from whom much which is of eternally practical value may not improbably be learnt. The thing above all others which the judicial mind would like to see, would be a concentration of all medical wisdom, ancient as well as modern, in such a convenient form that the man of the most limited reading might find himself at the bedside in a position to give every patient the best that medicine has to offer. To the majority of medical practitioners the famous and undoubtedly capable men of past generations are mere names, and to some hardly even so much as that. There is no profession in which what is derisively called "learning" is less thought of than in that of medicine. The ordinary practitioner prides himself above all things upon being what he calls "practical." But a man is practical who merely puts a nail through a plank on the ship's deck, and another is also practical who lays the plan and builds the ship. The one is a practical day labourer; the other a practical architect and man of science. The second year's student is practical when he puts a single stitch into a cut scalp, and the craniotomist is also practical when he trephines a skull, removes a bony plate, and cures a man of dull imbecility or dangerous convulsions by the operation. It is always the man of knowledge and of reasoning power who is most practical. The ignorant man whose boast is in his practicality is generally unpractical, and sometimes even dangerous in proportion to the measure of his ignorant boasting.

The fashion of the moment, in all the learned professions, is to avoid systematising, and to heap up details and criticisms. It is not a fashion that tends to develope either thinking power or practical capacity. If a man be not a theoriser he will not be a practiser of any merit. "Better is a bad theory than none," says Sir William Hamilton, "Happy is the man that has the best." What one would fain see, we repeat, is a consistent system of medical principles selected from the accumulations of medical knowledge and experience in all ages of which we have now such an abundance. Of Encyclopædias, Dictionaries, and Systems of Medicine in detail we have already enough, and, perhaps, almost more than enough. But of brief statements of general principles of medical science and practice, and of short catechisms, which set forth in dogmatic forms the main lines of such science and practice, without details, we have really none at all.

The inconsiderate man, the unreflecting, speaks slightingly of catechisms and dogmatic systems; and this he does because he is inconsiderate and unreflecting. For teaching purposes nothing whatever is so valuable as brief statements of comprehensive principles. Such statements act the part of well-constructed ships, into which an almost infinite variety and quantity of goods can be securely packed and carried with ease and safety across the most stormy seas. The want of such a system of compact principles is like throwing the same quantity and variety of goods into a harbour without a ship, and expecting them of their own accord to assort themselves and float out on the open sea to their different ports of destination. It is easy to imagine what a collapse of commerce would ensue if, instead of building ships for safe transit, every merchant should cast his merchandise indiscriminately into the sea.

But how would it be possible, asks the reader, impressed with the number and variety of medical and scientific treatises, to reduce to order a chaos which has, perhaps, never been equalled for confusion since the world began? It is precisely because the chaos is so overwhelming that light and order should now be invoked. Are we to admit that there are no well-defined principles of medical knowledge and practice which can be clearly and authoritatively set forth? Every intelligent physician knows that there are such luminous principles, that there are competent expounders of medical science who could set them forth in due measure and with illuminating clearness, and that such a body of principles would be invaluable, not only for the student, but for every practitioner of medicine who has the slightest desire to practice his art philosophically and scientifically. It is decidedly a peculiar circumstance that no such brief exposition of principles has ever been published; and it demands an explanation. The explanation is probably threefold. In the first place the task, to be properly accomplished, is a very great one; and if it were not properly accomplished it would be better left alone. The second reason is that the half-dozen or less who could best do it are more profitably employed, that is, from a financial point of view; and the third, that a large part of the medical profession does not appear to be intelligent enough to be conscious of the need of a luminous exposition of principles. But if the writer were found who could and would give us such an exposition in brief and adequate form, he would confer greater scientific and practical advantages upon the working members of the profession than any other man of his generation.

